# Media and technology usage and attitudes in emergency department patients

**DOI:** 10.3389/fdgth.2022.894683

**Published:** 2022-10-31

**Authors:** C. E. Goldfine, A. Knapp, G. R. Goodman, M. A. Hasdianda, H. Huang, A. D. Marshall, Y. G. Keschner, S. Carreiro, G. Jambaulikar, P. R. Chai

**Affiliations:** ^1^ Department of Emergency Medicine, Brigham and Women's Hospital, Boston, MA, United States; ^2^ The Fenway Institute , Boston, MA, United States; ^3^ The Koch Institute for Integrated Cancer Research, Massachusetts Institute of Technology, Boston, MA, United States; ^4^ Massachusetts General Hospital, Springboard Studio, Boston, MA, United States; ^5^ Department of Emergency Medicine, University of Massachusetts Medical School, Worcester, MA, United States; ^6^ Department of Psychosocial Oncology and Palliative Care, Dana Farber Cancer Institute, Boston, MA, United States

**Keywords:** technology, media, smartphones, mobile health, digital health

## Abstract

**Introduction:**

Digital health technologies are increasingly being used in emergency medicine, many of which utilize smartphones and computers. Patient willingness to use these modalities is an important factor in successful implementation. Therefore, this study aimed to assess emergency department (ED) patients' use of and attitudes towards technology.

**Methods:**

This was a pooled sub-analysis of ED patients (≥18 years old) that were enrolled in two studies evaluating the ED patient experience in response to novel technological interventions. Participants completed the Media and Technology Usage and Attitudes Scale (MTUAS) that assessed computer and smartphone ownership; frequency of use of phone calls, texting, email, and smartphones; and anxiety and dependence attitudes on these technologies.

**Results:**

One hundred and forty-four participants completed the survey. Mean age was 47.2 years (SD 17.94); 61.8% were female; and 61.1% were white. There was high usage of smartphones (93.1%) and computers (74.3%). Participants most frequently used phone calling and texting and least commonly used email. Participants had a positive attitude (mean 3.9/5, SD 0.68) towards the use of these technologies.

**Discussion:**

ED patients reported high ownership of smartphones and computers, had a positive attitude towards their use, and had varying frequency with which they used different technologies. Future studies can use this information to inform the development of digital health interventions that utilize technologies that patients find most acceptable.

## Introduction

Over the past two decades, mobile technologies have become increasingly popular. The introduction of smartphones now enables individuals to be connected to the internet on a nearly continuous basis, to interact and receive messages from a variety of social networks, and to seamlessly record real-world events on demand. In 2021, a longitudinal study showed that 97% and 85% of Americans today own cell phones or smartphones, respectively, a significant increase from 2011, when 86% of individuals owned cellular phones and only 35% owned smartphones (1).

In parallel with this increase in smartphones, as well as other wearable and mobile technologies, has been the emergence of digital health and wellness related interventions. Wearable devices that track physical activity and fitness, smartwatches, and other digitized devices now permit the collection of a wide variety of both physiologic and digital phenotypic data. This information can, in turn, inform the deployment of an array of behavioral health interventions with the potential to influence and improve the management of chronic disease antecedent to exacerbations of illness. For example, behavioral interventions linked to real-time medication adherence data, collected *via* ingestible sensors embedded within a prescribed medication, may aid in addressing medication nonadherence earlier, and more directly, than was previously possible (2). Similarly, data collected by wearable sensors may indicate physiological signs of substance use in settings previously inaccessible by clinicians, thereby enabling prompt intervention (3).

With the increased emphasis on digital health interventions in both clinical and research contexts, the emergency department (ED) represents an key context for the deployment of such interventions; existing digital health data from the ED may additionally be queried to inform clinical decision making in acute illness. Previous investigations have indicated that patients in the ED possess smartphones at an equivalent rate to the general US population, and are willing to utilize them for health-related tasks (4). Some digital health interventions leverage the ED setting to address not only acute complaints, but post-discharge care prior to linkage to a patient's primary providers (5, 6). Despite the deployment of these interventions, little is known about ED patients' baseline attitudes and utilization of technologies outside of smartphones. In response, we utilized the Media and Technology Usage and Attitudes Scale (MTUAS), a validated measure to assess both usage of technologies as well as baseline attitudes towards technologies, among ED patients who were participating in ED-based digital health clinical trials.

## Methods

This is a pooled sub-analysis of *N* = 144 participants who enrolled in two separate prospective observational cohort studies addressing technological interventions to influence the ED patient experience at an academic, urban, tertiary-care level medical center with more than 65,000 annual emergency department visits in Boston, MA (7, 8). The two respective studies were approved by the Mass General Brigham Institutional Review Board.

We enrolled a convenience sample of ED patients over the age of 18 years who met the eligibility criteria for one of the studies. Participants in both studies were English-speaking adults triaged to private rooms in the ED who were able to complete quantitative assessments and who did not require acute emergent medical intervention; additional eligibility criteria and enrollment information is reported within the two parent studies (7, 8). Enrolled participants were asked to complete the Media and Technology Usage and Attitudes Scale (MTUAS) at their baseline assessment (9). The MTUAS version used in these studies consisted of 32 items across the (1) emailing, (2) smartphone, (3) phone calling, and (4) texting usage subscales, as well as the (5) positive and (6) anxiety/dependence attitudes scales (Appendix 1). The four Usage subscales used a 10-item frequency response scale (never, once a month, several times a month, once a week, several times a week, once a day, several times a day, once an hour, several times an hour, and all the time) (9). The two Attitudes subscales assessed participants' attitudes toward media and technology *via* a five-point Likert scale (strongly agree, agree, neither agree nor disagree, disagree, and strongly disagree). Quantitative data related to smartphone and computer ownership was also collected. MTUAS data was collected and managed using the Research Data Electronic Capture (REDCap) tools hosted at Mass General Brigham (10, 11).

## Results

Over the study periods for the two studies, a total of 216 eligible ED patients were approached. Seventy-two patients declined to participate in the studies for the following reasons: no interest in research (*n* = 30, 41.7%), too tired (*n* = 16, 22.2%), too sick (*n* = 9, 12.5%), privacy concerns (*n* = 4, 5.6%), and other reasons (*n* = 13, 18.1%). One hundred and forty-four participants from both studies completed the MTUAS survey. The majority of the sample was female (*n* = 89, 61.8%) and 61.1% were White (*n* = 88) (Table 1). The average age was 47.2 years old [standard deviation (SD) ± 17.94].

**Table 1:** Participant demographics.

Age, years (mean, SD)	47.2 (17.94)
Race (*n*, %)	
Asian	7 (4.9)
Native Hawaiian/Pacific Islander	1 (0.7)
Black	34 (23.6)
White	88 (61.1)
Unavailable	14 (9.7)
Ethnicity (*n*, %)	
Hispanic/Latino	14 (9.7)
Non-Hispanic/Non-Latino	125 (86.8)
Unavailable	5 (3.5)
Female (*n*, %)	89 (61.8)

The majority of participants owned smartphones (93.1%, *n* = 134) and computers (74.3%, *n* = 107), (Table 2). Participants reported using phone calls and texting several times a day. Smartphones were used about once a day. Email use was the least frequent, with reported use ranging from several times a week to once a day. Participants reported having a positive attitude (mean 3.9/5, SD 0.68) towards phone calls, texting, smartphones, and email. However, they neither agreed nor disagreed that they felt anxiety if they could not use these technologies, and neither agreed nor disagreed that they were dependent on these technologies (mean 3.2/5, SD 0.68).

**Table 2:** MTUAS results.

Device Ownership (*n*, %)	
Smartphone	134 (93.1)
Laptop/Computer	107 (74.3)
MTUAS Usage Subscales (1–10)^a^	
Emailing	5.9 (2.7)
Phone calling	7.4 (1.87)
Texting	7.2 (1.97)
Smartphone usage	6.4 (2.24)
MTUAS Attitudes Subscales (1–5)^b^	
Positive attitude	3.9 (0.68)
Anxiety and dependence attitude	3.2 (0.68)

^a^ 1, Never | 2, Once a month | 3, Several times a month | 4, Once a week | 5, Several times a week | 6, Once a day | 7, Several times a day | 8, Once an hour | 9, Several times an hour | 10, All the time.

^b^ 1, Strongly Disagree | 2, Disagree | 3, Neither Agree nor Disagree | 4, Agree | 5, Strongly Agree

## Discussion

The ED represents a unique environment for the implementation of digital health technologies and associated interventions. Patients often present to the ED due to concerns surrounding critical illnesses, including heart attack, stroke, or trauma. Prior studies have demonstrated that these vulnerable moments can function as opportunities when patients are ready to make changes and implement interventions that can improve their health (12–14). With increasing adoption of technologies such as smartphones, the use of digital health interventions may be attractive options for addressing not only emergency care but post-ED care as well. This investigation demonstrated that there continues to be widespread uptake of smartphones and technologies among ED patients, and that attitudes towards using technology are positive. This suggests that continued research and development of digital health interventions, which utilize technologies that already have a high level of patient acceptability, may be adopted by ED patients.

In our study of 144 ED patients, there was high ownership of both smartphones (93.1%) and computers (73.3%). The most used technology was phone calling and texting, followed by smartphone usage, and emailing. Overall, participants had a positive view of such technologies. These data demonstrate that the ED may be a novel location for digital health interventions and that, despite the acute illnesses that may prompt individuals to seek emergency care, their attitudes towards technologies may provide an opportunity to deploy technology-based interventions.

The ability to provide digital health technologies to ED patients has a variety of implications given the myriad medical issues that lead to ED presentations. Technologies that enhance outpatient monitoring could provide the ability to care for patients in settings that were previously inaccessible; the detection of acute exacerbations of chronic conditions could lead to interventions prior to severe decompensation; and real-time detection of at-risk behaviors could provide opportunities for interventions aimed at behavioral modifications. In addition to providing support for the use of digital health interventions, this study also presents unique findings that can help guide the development of future digital health interventions. Specifically, understanding the frequency of use of specific technologies can aid in informing the modalities that would be most useful and acceptable depending on intervention type and goals. For example, as many patients do not check their email on a daily basis, an intervention that requires daily check-ins or behavioral health modifications, such as real-time adherence reminders, may have better uptake with texting, rather than email, as patients tend to use this technology more frequently.

Our findings were similar to a 2012 study of ED patients that evaluated their willingness to use technologies for behavioral interventions (15). In that study, patients that presented to an urban tertiary care ED also had high baseline use of technologies and were interested in the use of technology for behavioral health interventions. As digital health technology is a rapidly advancing field, it is important to evaluate how patients' perceptions and acceptance of specific technologies change over time. Our study adds to the body of work showing that healthcare technologies that utilize smartphones or computers could be feasible for use by ED patients given the high current usage and ownership of these modalities.

There were some limitations in this study. First, this was a quantitative survey, and therefore we did not obtain any qualitative data that may have added additional depth to our interpretation of findings. This was also a single-site study in a large urban environment, which may limit its generalizability to ED patients in other geographical contexts. Additionally, the survey was only available in English, and therefore, non-English speakers were excluded which may have unintentionally missed populations less comfortable with technology. Finally, this was a sub-analysis from two larger studies, both of which evaluated the implementation of novel technologies in the ED. This may have led to a bias among participants, who may have been more accepting of advanced technology than individuals who were not enrolled in those studies.

In order for new technologies to be successfully used in healthcare settings, patients must be willing and accepting of their use. This study provides important information as technology is increasingly being leveraged, especially during the Coronavirus pandemic, where in-person contact has been limited for safety and telemedicine and mobile health technologies are being relied upon, not only for at-home monitoring but also healthcare and wellness interventions. Additionally, the ability to provide real-time interventions and feedback to patients can have large implications for improving longitudinal care and support for patients at vulnerable times. Future studies can use the results of this study to inform the development and implementation of new technologies on platforms that patients are comfortable and accepting of using.

## Data Availability

The raw data supporting the conclusions of this article will be made available by the authors, without undue reservation.

## APPENDIX 1

Part A: The media and technology usage and attitudes scale (MTUAS) questions

1. Do you own a smartphone? Yes or No
2. Do you own a laptop, or desktop computer? Yes or No
  * If no to question 1, complete sections 3 and 5
  * If no to question 2, complete sections 4 and 5

For the following questions use the following 10 item frequency scale:

*Never (1)*
*Once a month (2)*
*Several times a month (3) Once a week (4)*
*Several times a week (5) Once a day (6)*
*Several times a day (7) Once an hour (8)*
*Several times an hour (9) All the time (10)*

3. Please indicate how often you do the following email activities on your laptop or desktop?
  1. Send, receive and read e-mails: 1 2 3 4 5 6 7 8 9 10
  2. Check your personal 1 2 3 4 5 6 7 8 9 10
  3. Check your work of school 1 2 3 4 5 6 7 8 9 10
  4. Send or receive files *via* 1 2 3 4 5 6 7 8 9 10
4. Please indicate how often you do each of the following activities on your mobile phone.
  1. Send and receive text messages on a mobile phone: 1 2 3 4 5 6 7 8 9 10
  2. Make and receive mobile phone calls: 1 2 3 4 5 6 7 8 9 10
  3. Check for text messages on a mobile phone: 1 2 3 4 5 6 7 8 9 10
  4. Check for voice calls on a mobile phone: 1 2 3 4 5 6 7 8 9 10
  5. Read email on a mobile phone: 1 2 3 4 5 6 7 8 9 10
  6. Get directions or use GPS on a mobile phone: 1 2 3 4 5 6 7 8 9 10
  7. Browse the web on a mobile phone: 1 2 3 4 5 6 7 8 9 10
  8. Listen to music on a mobile phone: 1 2 3 4 5 6 7 8 9 10
  9. Take pictures using a mobile phone: 1 2 3 4 5 6 7 8 9 10
  10. Check the news on a mobile phone: 1 2 3 4 5 6 7 8 9 10
  11. Record video on a mobile phone: 1 2 3 4 5 6 7 8 9 10
  12. Use apps (for any purpose) on a mobile phone: 1 2 3 4 5 6 7 8 9 10
  13. Search for information with a mobile phone: 1 2 3 4 5 6 7 8 9 10
  14. Use your mobile phone during class or work time: 1 2 3 4 5 6 7 8 9 10
5. For the next series of statements use the following scale:
  *Strongly Agree (5)*
  *Agree (4)*
  *Neither Agree nor Disagree (3) Disagree (2)*
  *Strongly Disagree (1)*
  1. I feel it is important to find any information whenever I want online: 1 2 3 4 5
  2. I feel it is important to be able to access the Internet any time I want: 1 2 3 4 5
  3. I think it is important to keep up with the latest trends in technology: 1 2 3 4 5
  4. I get anxious when I don't have my cell phone: 1 2 3 4 5
  5. I get anxious when I don't have the internet available to me: 1 2 3 4 5
  6. I am dependent on my technology: 1 2 3 4 5
  7. Technology will provide solutions to many of our problems: 1 2 3 4 5
  8. With technology, anything is possible: 1 2 3 4 5
  9. I feel I get more accomplished because of technology: 1 2 3 4 5
  10. New technology makes people waste too much time: 1 2 3 4 5
  11. New technology makes life more complicated: 1 2 3 4 5
  12. New technology makes people more isolated: 1 2 3 4 5
